# The antibacterial activity and mechanism of imidazole chloride ionic liquids on *Staphylococcus aureus*

**DOI:** 10.3389/fmicb.2023.1109972

**Published:** 2023-02-06

**Authors:** Yanhui Hu, Yuyuan Xing, Peng Ye, Haikuan Yu, Xianglei Meng, Yuting Song, Gongying Wang, Yanyan Diao

**Affiliations:** ^1^Chengdu Institute of Organic Chemistry, Chinese Academy of Sciences, Chengdu, China; ^2^Beijing Key Laboratory of Ionic Liquids Clean Process, CAS Key Laboratory of Green Process and Engineering, State Key Laboratory of Multiphase Complex Systems, Institute of Process Engineering, Chinese Academy of Sciences, Beijing, China; ^3^University of Chinese Academy of Sciences, Beijing, China; ^4^Innovation Academy for Green Manufacture, Chinese Academy of Sciences, Beijing, China; ^5^Beijing Key Laboratory of Lignocellulosic Chemistry, Beijing Forestry University, Beijing, China; ^6^Senior Department of Orthopedics, Chinese PLA Medical School, Beijing, China

**Keywords:** ionic liquids, *Staphylococcus aureus*, antibacterial activity, mechanism, skin abscess

## Abstract

Ionic liquids (ILs) have garnered increasing attention in the biomedical field due to their unique properties. Although significant research has been conducted in recent years, there is still a lack of understanding of the potential applications of ILs in the biomedical field and the underlying principles. To identify the antibacterial activity and mechanism of ILs on bacteria, we evaluated the antimicrobial potency of imidazole chloride ILs (C_n_MIMCl) on *Staphylococcus aureus* (*S. aureus*). The toxicity of ILs was positively correlated to the length of the imidazolidinyl side chain. We selected C_12_MIMCl to study the mechanism of *S. aureus*. Through the simultaneous change in the internal and external parts of *S. aureus*, C_12_MIMCl caused the death of the bacteria. The production of large amounts of reactive oxygen species (ROS) within the internal parts stimulated oxidative stress, inhibited bacterial metabolism, and led to bacterial death. The external cell membrane could be destroyed, causing the cytoplasm to flow out and the whole cell to be fragmented. The antibacterial effect of C_12_MIMCl on skin abscesses was further verified *in vivo* in mice.

## 1. Introduction

In recent years, diseases caused by bacteria have plagued human beings. Although antibiotics have alleviated this problem, excessive use also causes problems such as bacterial resistance. Considering the overuse of antibiotics and the increasing resistance to antibacterial agents, there is an urgent need to develop tunable antibacterial compounds and drug delivery systems to treat high-mortality diseases. Moreover, the COVID-19 pandemic has garnered more attention than ever regarding the need for more effective medical methods to diagnose and cure the disease. New, high-performing antimicrobial and antiviral materials have to be developed. As a combination of salt and organics, ionic liquids (ILs) have been endowed with abundant and diverse properties (Zhang et al., 2014), including low volatility, a wide electrochemical window, a highly tunable structure, and thermal stability, which have been widely used as solvents (Liang et al., 2019; Shamshina and Berton, 2020), extractants (Gao et al., 2020; Wang et al., 2022), catalysts (Li et al., 2021; Sadjadi, 2021; Yuan et al., 2023), electrolytes (Fu et al., 2022), and so on. ILs could be self-assembled into low-dimensional nanoparticles due to their strong interactions between the cations and anions, H-bond direction, and large molecular volume (Dupont, 2011; Chen et al., 2014). Their unique properties have attracted increasing attention in the biomedical field, such as the extraction and conservation of proteins (Schröder, 2017; Veríssimo et al., 2021; Xue et al., 2022) and nuclear acids (Dinis et al., 2020), drug transport enhancers (Md Moshikur et al., 2020; Zhang et al., 2020), drug carriers (Lu et al., 2020; Moshikur et al., 2021), drug additives (Tang et al., 2016), and disease diagnosis (Zhu et al., 2020) and therapy (Albadawi et al., 2021; Gao et al., 2021). The nanoparticle structure of ILs provides a variety of possibilities for biomedical applications. *Staphylococcus aureus* is a common foodborne pathogenic gram-positive bacterium which can cause a variety of serious infections (e.g., pneumonia, enteritis, pericarditis, and sepsis) in humans and animals through the skin and food (Shangguan et al., 2015; Nithya and Sundrarajan, 2020). In the treatment process, *S. aureus* has strong resistance and low susceptibility to drugs; thus, it is urgent to develop new ingredients to counteract the problem of drug resistance (Hess et al., 2005). In this study, *S. aureus* was selected to study the toxicity and mechanism of ILs on the bacterium.

Although ILs have been extensively studied in various directions for biomedical applications (Egorova et al., 2017), they currently remain in the preliminary stage due to a lack of systematic research and theoretical basis. In addition, there is little relevant research on ILs as an agent *in vivo*. An exciting avenue for research is to determine whether ILs can be used in developing antibacterial drugs due to their wide variety, adjustable structure, excellent properties, and the mechanism by which they interact with bacteria. Florio et al. (2019) compared and evaluated the antimicrobial efficacy of 15 ILs, including 1-methyl-3-dodecylimidazolium bromide, 1-dodecyl-1-methylpyrrolidinium bromide, and 1-dodecyl-1-methylpiperidinium bromide, which had strong inhibitory effects on the biofilm formation of *S. aureus* or *Pseudomonas aeruginosa* (*P. aeruginosa*) (Florio et al., 2019). Brunel et al. (2016) found that triphenylamine phosphonium ILs could self-assemble into nanoparticles, which have a good antibacterial effect on *S. aureus*. The IL nanoparticles were likely to strongly affect bacterial metabolism (Brunel et al., 2016). Although some researchers have speculated on the mechanism by which ILs interact with *S. aureus*, it remains unknown how ILs affect bacterial membranes and influence bacterial metabolism. Further studies are needed to better understand the mechanisms involved in antibacterial activity.

Because imidazole chloride ILs are widely available and studied in a variety of fields, a series of C_n_MIMCl were chosen to investigate toxicity mechanisms on bacteria and gain additional insight into the effects of IL structure on antibacterial properties. This study explored the toxicity and mechanism of ILs in gram-positive bacteria (*S. aureus*) and designed and developed ILs for infection prevention and control. A skin abscess model was established to demonstrate the antimicrobial efficacy of ILs *in vivo*.

## 2. Materials and methods

### 2.1. Strains, culture conditions, and ILs

The *S. aureus* BNCC 186335 and USA300-sfgfp strains were purchased from Forhigh Biotech. The *S. aureus* strain was cultivated in LB medium (18 g/L Nutrient Broth (NB), Solarbio) for 24 h at 37°C and 180 r/min in a shaker incubator. A microplate reader (Tecan, Infinite M2000) was used to measure OD_600_. ILs (C_2_MIMCl, C_3_MIMCl, C_4_MIMCl, C_6_MIMCl, C_8_MIMCl, C_12_MIMCl, C_16_MIMCl, ≥99%) were prepared by Shanghai Cheng Jie Chemical Co., Ltd. Then, the ILs were diluted in sterile, distilled water. Cy5 NHS Ester (Cy5, SE) was purchased from Beijing Fanbo Biochemicals Co., Ltd. Then, 4% of the paraformaldehyde fix solution was purchased from Solarbio.

### 2.2. Antibacterial assay

Iconic liquids were diluted in sterile, distilled water following the corresponding concentration gradient. Then, 50 μL of bacteria solutions and different ILs were added to 5 ml of LB medium. Afterward, mixed solutions were hatched at 37°C and 180 r/min in a shaker incubator for 24 h. Before measuring OD_600_, the solution was fully shaken to distribute *S. aureus* and ensure the accuracy of the measurement. Finally, 100 μL of solutions were removed from each sample and placed into 96-well plates to measure OD_600_. To avoid human or machine error, every OD_600_ value was repeated at least three times. EC50s were determined from OD_600_ by GraphPad Prism8. The activity of *S. aureus* cultured with ILs was observed by a confocal microscope, and the fluorescence was quantified.

Then, 200 μL of IL was added to 10 mL of solid LB medium (18 g/L NB, 1L water, and 15 g agarose), which was melted to liquid in advance and put in 10-cm plates. Then, 100 μL of bacterial suspension (OD_600 =_ 0.15) with further dilution was spread on solid LB plates and cultured at 37°C for 48 h. The CFUs were counted after being cultured, and the cultures were photographed with a camera.

### 2.3. Oxidative stress in *S. aureus*

C_3_MIMCl or C_12_MIMCl was added to *S. aureus* (OD_600_ = 0.15) and mixed with the ROS Assay Kit (DCFH-DA), and the mixed solution was placed in a 96-well plate for 30 min at 37^o^C. Then, the ROS value was detected with the microplate reader at the excitation wavelength of 488 nm and the emission wavelength of 525 nm. Following the above experimental procedure, 200 μL of the mixture was added to a confocal dish, and fluorescence was photographed using confocal laser scanning microscopy (Nikon Corporation, A1, CLSM) at the excitation wavelength of 488 nm with green as the pseudo color.

### 2.4. Surface morphology characterization and status observation of *S. aureus*

*Staphylococcus aureus* (OD_600_ = 0.15) was cultured in a liquid LB medium with ILs within 3 h at 37°C. Then, the bacteria were washed with PBS three times and fixed in 4% of the paraformaldehyde fix solution at 4°C overnight. The next day, bacteria were washed with PBS three times and treated with ethanol gradient dehydration at different concentrations (30, 40, 50, 60, 70, 80, 90, 95, and 100%). Bacteria were successfully mixed with different ethanol solution concentrations, were left to stand, were dehydrated for 15 min, and were collected by centrifugation. Finally, the bacteria dehydrated with 100% ethanol onto the tin foil was dropped and dried at 37°C for 2 h. The morphological changes of *S. aureus* treated with ILs were characterized by scanning electron microscopy (SEM, HITACHI, SU8020).

*S. aureus* was cultured with ILs, centrifuged (8000 r/min, 8 min), washed with water (3 times), and fixed with glutaraldehyde solution (PH = 7.4) overnight. They were taken in cross-sections and stained for further transmission electron microscopy (TEM, HITACHI, H-7650B) observation.

### 2.5. Measurement of *S. aureus* surface zeta potential

*S. aureus* (OD_600_ = 0.25) was treated with C_3_MIMCl and C_12_MIMCl (the final concentrations: 0.01 mM, 0.04 mM, and 0.16 mM) in liquid LB medium at 37°C for 3 h with gentle shaking. Then, *S. aureus* was washed with PBS three times and sterilized with water one time a day. The obtained bacteria were dispersed in 1 mL of water. The uniform suspension was measured for zeta potential using a Zetasizer Nano ZS (Malvern Instruments Ltd., Zetasizer Nano ZS90). As a control group, the bacteria were treated with an LB medium containing corresponding volumes of water at the same conditions. At the same time, we used Cy5, SE labeled C_12_MIMCl to interact with *S. aureus* and CLSM to observe the interaction between IL and bacteria.

### 2.6. Mice

BALB/c mice (6–8 weeks old, female) were obtained from Vital River Laboratories (Beijing, China). The Institutional Animal Care and Use Committees approved the animal protocol of the Institute of Process Engineering, Chinese Academy of Sciences (approval ID: IPEAECA 2022103). This study was performed in strict accordance with the Regulations for the Care and Use of Laboratory Animals and the Guideline for the Ethical Review of Animals.

### 2.7. Skin abscess model and antibacterial activity of ionic liquid *in vivo*

For the skin abscess model, mice were divided into three groups (controls, infected group, and ILs). Mice were anesthetized with pentobarbital sodium (1%) and inoculated with 100 μl of PBS containing 10^13^ cfu/L *S. aureus* or sterile PBS in the right flank by subcutaneous injection. After abscess formation, 50 μl of C_12_MIMCl (2 mM) was injected daily at the abscess site for 3–4 days. We observed the abscess healing continuously. Skin biopsy samples collected on day 11 were fixed and prepared for histopathological evaluation. The tissues were stained with hematoxylin and eosin (H&E) and 4′,6-diamidino-2-phenylindole (DAPI) and visualized by the scanner (3DHISTECH, P250 FLASH).

## 3. Results and discussion

### 3.1. Antibacterial activities of C_n_MIMCl against *S. aureus*

**Figure 1:**
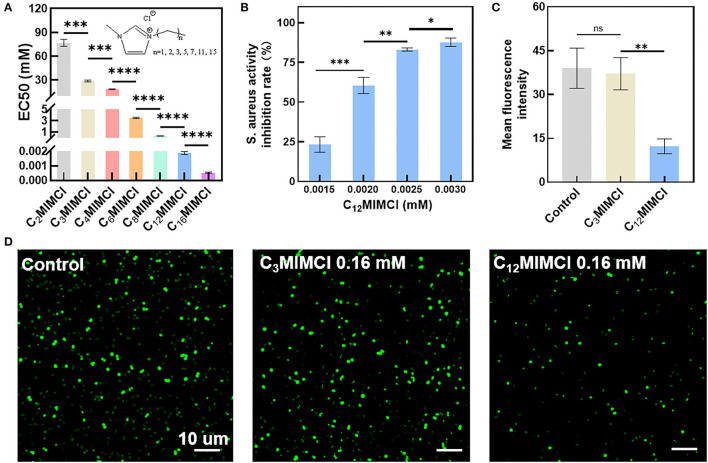
Toxicity test of C_n_MIMCl against *Staphylococcus aureus*. **(A)** EC50 values of *Staphylococcus aureus* incubated with C_n_MIMCl for 24 h. **(B)** Effects of C_12_MIMCl on *Staphylococcus aureus* at different concentrations. **(C)** Quantitative analysis of bacterial activity. **(D)** CLSM images of bacterial activity. Data in **(A–C)** represent the mean ± s.d. Statistical significance was calculated *via* two-tailed unpaired Student's *t*-test **(A–C)**. Ns means no significant difference, ^*^P<0.05, ^**^P < 0.01, ^***^P < 0.001, ^****^P < 0.0001.

The antibacterial activities of a series of imidazole chloride ILs against *S. aureus* were determined. To evaluate different ILs' antimicrobial activity against *S. aureus*, different concentrations of six ILs were used to measure EC50 (Figure 1A). The EC50 values in the LB medium of C_2_MIMCl, C_3_MIMCl, C_4_MIMCl, C_6_MIMCl, C_8_MIMCl, C_12_MIMCl, C_16_MIMCl were 76.7, 28.7, 18.2, 3.5, 0.3, 1.9 x 10^−3^, and 0.5 x 10^−3^ mM for *S. aureus*, respectively. Imidazolium chloride ILs with substituents of 12 and 16 carbon lengths had strong antibacterial effects on *S. aureus*. The toxicity of *S. aureus* increased with the length of the cation side chain. It may be due to the increased hydrophobicity. With an increase in C_n_MIMCl concentration, the inhibition rate of *S. aureus* increased. For example, when the concentration of C_12_MIMCl was 0.0015, 0.0020, 0.0025, and 0.0030 mM, the inhibition rates of *S. aureus* were 23.2, 60.5, 83.1, and 87.7%, respectively (Figure 1B). It showed that the toxicity of C_n_MIMCl varied for each concentration. *S. aureus* transfected with a green fluorescent protein (GFP) was incubated with C_3_MIMCl or C_12_MIMCl and observed by CLSM. There was no difference in GFP fluorescence intensity between the C_3_MIMCl group and the control group, while *S. aureus* GFP fluorescence intensity in the C_12_MIMCl group was significantly reduced (Figures 1C, D).

**Figure 2:**
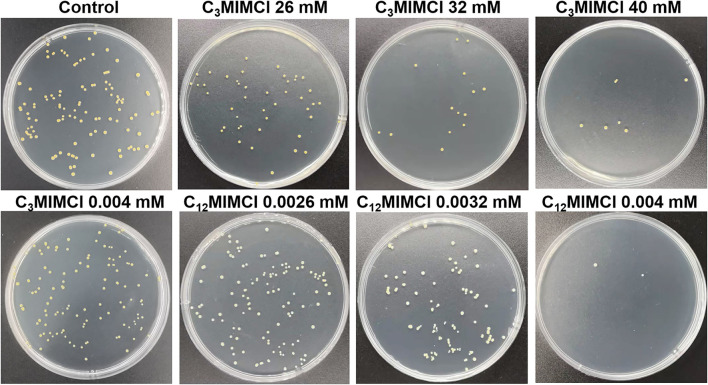
Bacterial colony photos of *Staphylococcus aureus* incubated with various concentrations of C_3_MIMCl and C_12_MIMCl for 48 h.

To further verify the toxicity of C_n_MIMCl, the less toxic C_3_MIMCl and the more toxic C_12_MIMCl were selected for plate coating experiments (Figure 2). Compared with the control group, there was no bacterial inhibition when the concentration of C_3_MIMCl was 0.0040 mM. The inhibition rates were 65.8 ± 0.8%, 89.1 ± 5.1%, and 94.6 ± 4.7% when the C_3_MIMCl concentration was 26 mM, 32 mM, and 40 mM, respectively. C_12_MIMCl showed a significant inhibitory effect on *S. aureus* compared with C_3_MIMCl. When the concentrations of C_12_MIMCl were 0.0026 mM, 0.0032 mM, and 0.0040 mM, the inhibition rates were 9.7 ± 3.9%, 23.0 ± 5.1%, and 98.4 ± 0.8%, respectively. With ~95% inhibition of *S. aureus*, the concentration used for C_3_MIMCl is about 10^4^ times higher than that used for C_12_MIMCl.

The toxicity of C_n_MIMCl to *S. aureus* was proportional to the length of the imidazolidinyl side chain. At the same time, the longer the alkyl side chain length of the imidazole, the greater the hydrophobicity of C_n_MIMCl. Therefore, the toxicity of C_n_MIMCl to *S. aureus* increased with the enhancement of hydrophobicity. It is well known that phospholipid is one of the main components of the cell membrane, and the outer surface of the cell membrane is electronegative. The ILs were easily adsorbed to the bacterial surface due to the positive charge of the cationic part, and then, the ILs interacted with the phospholipid bilayer due to hydrophobicity. Therefore, C_3_MIMCl with a short carbon chain and C_12_MIMCl with a long carbon chain were selected for the subsequent research.

### 3.2. Oxidative stress of *S. aureus* by C_12_MIMCl

**Figure 3:**
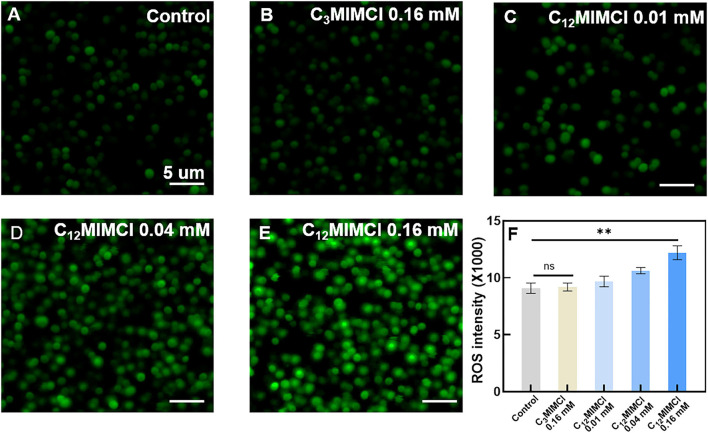
Oxidative stress of *Staphylococcus aureus*. **(A)** CLSM images of ROS release from bacteria in control. **(B–E)** CLSM images of ROS release from bacteria in the presence of 0.16 mM C_3_MIMCl **(B)**, 0.01 mM C_12_MIMCl **(C)**, 0.04 mM C_12_MIMCl **(D)**, 0.16 mM C_12_MIMCl **(E)**. **(F)** Quantitative analysis of ROS release by a microplate reader. Data in **(F)** represent the mean ± s.d. Statistical significance of **(F)** was calculated *via* two-tailed unpaired Student's *t*-test. Ns means no significant difference, **P < 0.01.

To further investigate the mechanisms leading to the inhibitory and destructive effects of C_12_MIMCl on *S. aureus*, as well as the significant reduction of the viability of the exposed bacteria induced by ILs, the release of reactive oxygen species (ROS) within *S. aureus* was explored. ROS is a general term for a class of molecules with oxidative activity produced by cells during energy metabolism under aerobic conditions. In bacteria, excess ROS can damage nucleic acids, proteins, and lipids (Brynildsen et al., 2013). Moreover, it can also lead to bacterial oxidative stress and inhibit bacterial metabolism (Ning et al., 2019; Zhao et al., 2022). Bacterial death occurs when intracellular ROS exceeds the cell's ability to detoxify and repair. Therefore, it is necessary to determine the amount of ROS in bacteria to reveal the mechanism of IL acting on bacteria. Figure 3 shows CLSM images of ROS (green). No significant changes in ROS fluorescence values were observed with 0.16 mM C_3_MIMCl compared with the control group. It was found that C_12_MIMCl could significantly increase ROS in bacteria compared with C_3_MIMCl at the same concentration, and the ROS gradually increased with an increase in C_12_MIMCl concentration. Therefore, C_12_MIMCl could induce ROS release intracellularly.

### 3.3. Damage to the *S. aureus* membrane by C_12_MIMCl

#### 3.3.1. Surface morphology observation of *S. aureus*

**Figure 4:**
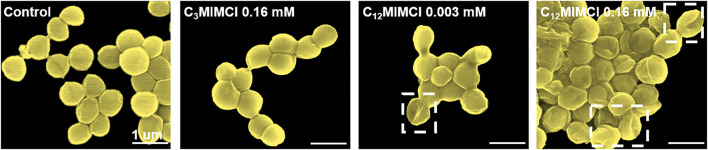
SEM characterization of *Staphylococcus aureus* morphologies and membrane integrities after 3h co-culture with water (control group), 0.16 mM C_3_MIMCl or different concentrations of C_12_MIMCl (0.003 mM and 0.16 mM).

SEM images were utilized to observe the morphological changes of *S. aureus* after incubation with ILs for 3 h. It could be seen that the normal form of *S. aureus* strains displayed regular and clear edges, smooth surfaces with rounded projections, and complete cell walls. To further investigate the antibacterial activities of short and long cation side chains, the damage of C_3_MIMCl and C_12_MIMCl on the bacterial membrane of *S. aureus* was evaluated (Figure 4). Furthermore, similar to their control counterparts, the morphologies of the bacteria treated with 0.16 mM C_3_MIMCl for 3 h remained intact and smooth. However, the morphology of *S. aureus* treated with C_12_MIMCl was collapsed by depression or wrinkling on the surface, indicating that partial or complete membrane lysis might occur. As the concentration of C_12_MIMCl increased, a greater effect on the surface morphology of *S. aureus* could be observed. This result indicated that C_12_MIMCl could damage the membranes of *S. aureus*.

#### 3.3.2. Changes on the *S. aureus* surface

**Figure 5:**
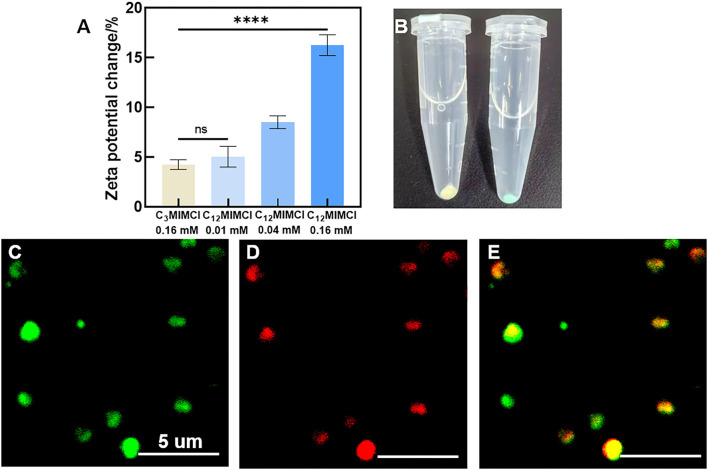
The changes of *Staphylococcus aureus* surface. **(A)** Zeta potentials of *Staphylococcus aureus* co-cultured with C_3_MIMCl or C_12_MIMCl in PBS for 3h. **(B)** Picture of *Staphylococcus aureus* incubated with Cy5, SE-C_12_MIMCl. **(C–E)** CLSM images of C_12_MIMCl interacting with *Staphylococcus aureus*. Green: *Staphylococcus aureus* transfected with GFP **(C)**, Red: Cy5, SE-C_12_MIMCl **(D)**, merged **(E)**. Data in **(A)** represent the mean ± s.d. Statistical significance of **(A)** was calculated *via* two-tailed unpaired Student's *t*-test. Ns means no significant difference, ****P < 0.0001.

Bacterial membrane potential is the proton motive force of bacterial metabolism and respiration. Antibacterial activity strongly correlates with the dissipation of the membrane potential in bacteria (Schilling et al., 2019). When studying the antibacterial mechanisms, the integrity of bacterial membranes is a non-negligible factor. In our study, when the concentration of C_12_MIMCl was 0.16 mM, C_12_MIMCl increased the bacterial membrane potential to 16.3%, and the change in membrane potential increased with an increase in ILs concentration. Compared with C_3_MIMCl, *S. aureus* showed an increase in zeta potentials after co-culturing with C_12_MIMCl (0.16 mM) (Figure 5A), indicating that the positively charged C_12_MIMCl underwent electrostatic interactions with the negatively charged components of the *S. aureus* membrane. With an increase in C_12_MIMCl concentration, the change in *S. aureus* membrane potential increased. The potential of *S. aureus* is in full correlation with their EC50 values and is concentration dependent. It could be seen that the bacterial color changed after incubation with Cy5, SE-C_12_MIMCl, indicating that C_12_MIMCl was adsorbed on the bacterial surface (Figure 5B). Because the *S. aureus* surface was negatively charged, it could attract the IL cation with a positive charge to gather on the bacterial surface by CLSM (Figures 5C–E). It was once again demonstrated that C_12_MIMCl could interact with bacterial cell membranes.

#### 3.3.3. Observation of the destruction process of S. aureus by ILs

**Figure 6:**
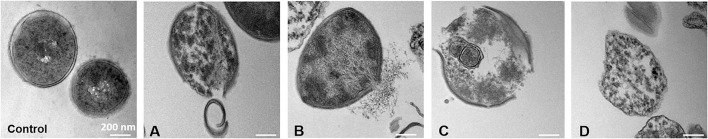
TEM images of *Staphylococcus aureus* within 3 h treatment with 0.16 mM of C_12_MIMCl. **(A-D)**: The destruction process of *Staphylococcus aureus* by C_12_MIMCl.

TEM images further confirmed that *S. aureus* was treated with C_12_MIMCl, which showed membrane detachment, content leakage, and cell disruption. Figure 6 shows the whole process of *S. aureus* being destroyed. The control group and C_3_MIMCl group showed intact and smooth cell membranes. Initially, part of the cell membrane was shed from the bacterial surface in the presence of C_12_MIMCl (Figure 6A). Subsequently, the cytoplasm flowed out from the broken cell membrane (Figure 6B). Then, the cell membrane of *S. aureus* was completely disrupted (Figure 6C). Finally, *S. aureus* was dead (Figure 6D).

### 3.4. Antibacterial mechanism of ionic liquids against *S. aureus*

**Figure 7:**
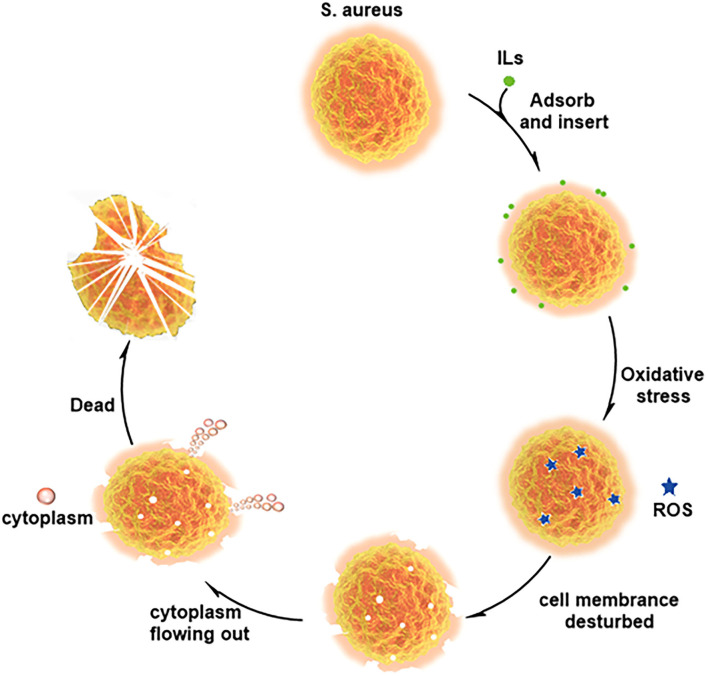
Plausible antibacterial mechanism for interaction between C_12_MIMCl and *Staphylococcus aureus*.

The cell membrane mainly consists of phospholipids with a negative charge on the surface and hydrophobicity. ILs are amphiphilic, and long cationic side chains can be inserted into the phospholipid bilayer, causing cell membrane disorder (Kaur et al., 2020; Liu et al., 2021). According to the above experimental contents, C_12_MIMCl could be adsorbed on the surface of *S. aureus* by electrostatic action. Afterward, C_12_MIMCl was inserted into cell membranes driven by hydrophobicity. This led to an oxidative stress reaction in bacteria, that is, the rapid production of a large amount of ROS. At the same time, it changed the permeability of the bacterial cell membrane, which could change the bacterial osmotic pressure, destroying the cell membrane and allowing cytoplasm to flow out of the broken cell membrane. Figure 7 depicts a plausible antibacterial mechanism for interacting C_12_MIMCl with *S. aureus*.

### 3.5. The therapeutic effect of C_12_MIMCl on skin abscess *in vivo*

#### 3.5.1. Observation of a skin abscess

**Figure 8:**
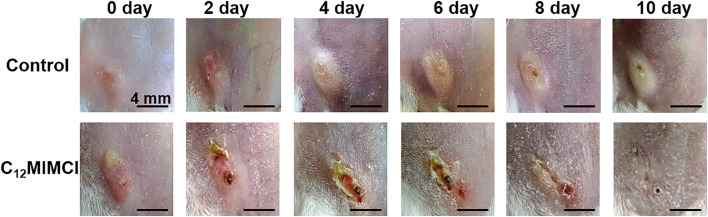
Changes of skin abscess in mice after *Staphylococcus aureus* infection and C_12_MIMCl treatment.

The skin of *S. aureus*-infected mice developed visible abscesses, redness, edema, and ulcers within 1–2 days of infection. The abscess was injected with 2 mM C_12_MIMCl (50 μl) for 3–4 days. On the second day of C_12_MIMCl administration, the abscess showed a distinct black scab, and the abscess under the skin gradually decreased, while the abscess in the control group gradually grew larger (Figure 8). In the following days, the scabbed area gradually expanded. The subcutaneous abscess did not disappear completely until 3–4 days after the C_12_MIMCl injection. After 8 days, the scab gradually fell off. The skin in the treatment group could be healed for 10–12 days. However, the subcutaneous abscess remained in the control group.

#### 3.5.2. Analysis of skin tissue

**Figure 9:**
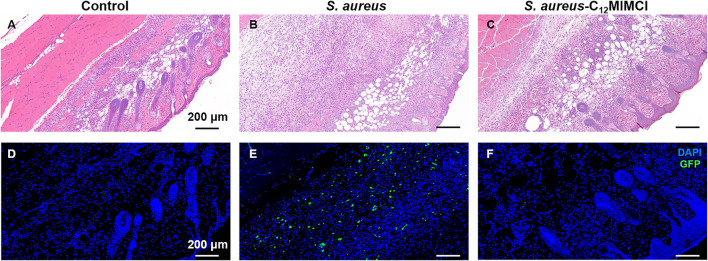
Histopathology of skin abscess and fluorescent scan in mice after *Staphylococcus aureus* infection and C_12_MIMCl treatment. **(A)** Histopathology of the control group (PBS). **(B)** Histopathology of skin abscess after *Staphylococcus aureus* injection. **(C)** Histopathology of C_12_MIMCl treatment in skin abscess. **(D)** Fluorescent scan of the control group (PBS). **(E)** Fluorescent scan of skin abscess after S*taphylococcus aureus* injection. **(F)** Fluorescent scan of C_12_MIMCl treatment in skin abscess. DAPI (blue), GFP*-Staphylococcus aureus* (green).

Skin biopsy samples collected on day 11 were fixed and prepared for histopathological evaluation (Figures 9A–C). Compared with the control group, the wavy curves of the epidermis and dermis at the subcutaneous abscess sites of *S. aureus* infection disappeared, indicating that the epidermis and dermis were severely damaged. In addition, there was a large infiltration of monocytes in the dermis. After the C_12_MIMCl injection, the morphology of the epidermis and dermis gradually recovered, and the number of inflammatory cells decreased significantly. H&E staining of skin sections from the mice revealed that C_12_MIMCl had a good therapeutic effect on the subcutaneous abscess. After DAPI staining and GFP spontaneous fluorescence scanning, it could be seen that the number of *S. aureus* in the skin was nearly zero after the C_12_MIMCl injection. The number of *S. aureus* cells in the skin remained largely unchanged in the untreated group (Figures 9D–F). In conclusion, C_12_MIMCl could improve the skin abscess infected with *S. aureus* in mice.

## 4. Conclusion

A variety of imidazole chloride ILs with varying length substituents was investigated for their antibacterial mechanisms against *S. aureus*. Imidazole chloride ILs containing twelve and sixteen carbon lengths had strong antibacterial and anti-biofilm activity against *S. aureus*. The results showed that the antibacterial efficiency of ILs could be improved by changing the alkyl chain length due to the tunable structure of ILs. The above research demonstrated that long cationic side-chain ILs effectively inhibited *S. aureus*. This study provided new ideas for creating new antibacterial drugs. ROS release tests confirmed that long cationic side-chain ILs have a strong impact on the metabolism of *S. aureus*. SEM and membrane potential test experiments revealed that ILs could collapse and damage the surface of *S. aureus*. TEM images clearly showed how C_12_MIMCl disrupts the cell membrane of *S. aureus*, causing the contents to leak. Ultimately, the antibacterial mechanism of C_12_MIMCl against *S. aureus* was proven. *In vivo* tests, C_12_MIMCl had significant antibacterial effects and accelerated the healing of skin abscesses. After the new coronavirus pandemic in 2019, it is urgent to develop new antibacterial and antiviral drugs. ILs have not been found to be appropriate drugs for clinical use. At present, we know that the toxicity of ILs to *S. aureus* depends on the length of cationic alkyl chain substituents. In the future, we will expand the types of organism models (e.g., Gram-negative bacteria, viruses, cells, and animals) and design specific properties for different diseases by regulating different types of ILs substituents.

## Data availability statement

The original contributions presented in the study are included in the article/supplementary material, further inquiries can be directed to the corresponding authors.

## Ethics statement

The animal study was reviewed and approved by the Institutional Animal Care and Use Committees at the Institute of Process Engineering, Chinese Academy of Sciences.

## Author contributions

YH, GW, and YD contributed to the conception and design of the study. YH, PY, and HY performed the experiments. YH, PY, and XM contributed significantly to data analyses. YH and YX organized the pictures of the experiment. YH wrote the manuscript. HY, YX, and YS revised the manuscript. GW and YD oversaw the completion of this study. All authors contributed to the article and approved the submitted version.
